# Crystal structure of 1,3-bis­(1*H*-benzotriazol-1-yl­meth­yl)benzene

**DOI:** 10.1107/S2056989016007805

**Published:** 2016-05-17

**Authors:** Mario A. Macías, Nelson Nuñez-Dallos, John Hurtado, Leopoldo Suescun

**Affiliations:** a Cryssmat-Lab/Cátedra de Física/DETEMA, Facultad de Química, Universidad de la República, Montevideo, Uruguay; b Grupo INTERFASE, Universidad Industrial de Santander, Carrera 27, Calle 9, Ciudad Universitaria, Bucaramanga, Colombia; c Departamento de Química, Universidad de los Andes, Carrera 1 No 18A-12, Bogotá, Colombia

**Keywords:** crystal structure, benzotriazole derivative, benzotriazolophanes, bis­(1*H-*benzotriazol-1-ylmeth­yl)arene ligands

## Abstract

The mol­ecular structure of the title compound, C_20_H_16_N_6_, contains two benzotriazole units bonded to a benzene nucleus in a *meta* configuration, forming dihedral angles of 88.74 (11) and 85.83 (10)° with the central aromatic ring and 57.08 (9)° with each other. The three-dimensional structure is controlled mainly by weak C—H⋯N and C—H⋯π inter­actions. The mol­ecules are connected in inversion-related pairs, forming the slabs of infinite chains that run along the [-110] and [110] directions.

## Abstract 2

The crystal structure of 1,3-bis­(1*H*-benzotriazol-1-ylmeth­yl)benzene shows an inter­esting three-dimensional assembly influenced by its non-planar mol­ecular conformation.

## Chemical context   

Bis(1*H-*benzotriazol-1-ylmeth­yl)arene compounds are used as precursors for the synthesis of benzotriazolophanes, a class of positively charged cyclo­phanes that have the potential ability to trap anions and guest mol­ecules with high electron density (Rajakumar & Murali, 2000 ▸). On the other hand, the study of the self-assembly of helicates from the reaction of metal ions with bis­(1*H*-benzotriazol-1-ylmeth­yl)arene ligands has been of great inter­est. In these complexes, the metal center coord­inates through the N3-nitro­gen of the benzotriazole ring (O’Keefe & Steel, 2000 ▸). We have been inter­ested in the synthesis of metal complexes with ligands derived from benzotriazole, which show high activity as catalysts for oxidative amination of allyl butyl ether (Hurtado *et al.*, 2013 ▸).The crystal structures for a number of bis­(1*H-*benzotriazol-1-ylmeth­yl)arene ligands have been determined: 2,6-bis­(1*H*-benzotriazol-1-ylmeth­yl)pyridine (Selvanayagam *et al.*, 2002 ▸), 1,4-bis­(1*H*-benzotriazol-1-yl­meth­yl)­benzene tetra­hydrate (Cai *et al.*, 2004 ▸) and benzyl 3,5-bis­(1*H*-benzotriazol-1-yl­meth­yl)­phenyl ether (Selvanayagam *et al.*, 2004 ▸). As part of structural studies of the self-assembly process of metal ions with ligands derived from benzotriazole, we report here the crystal structure of the ligand 1,3-bis­(1*H*-benzotriazol-1-yl­meth­yl)benzene.
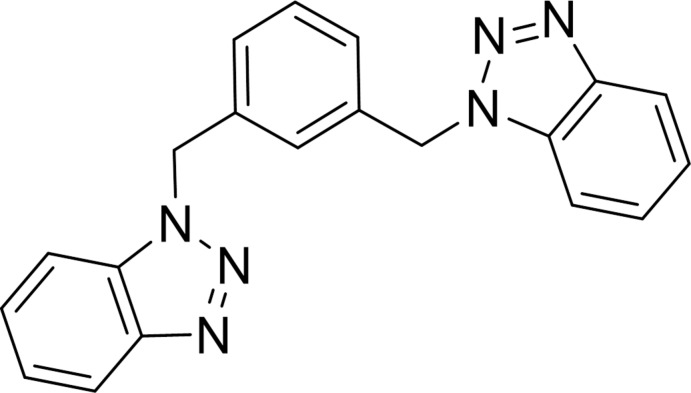


## Structural commentary   

**Figure 1:**
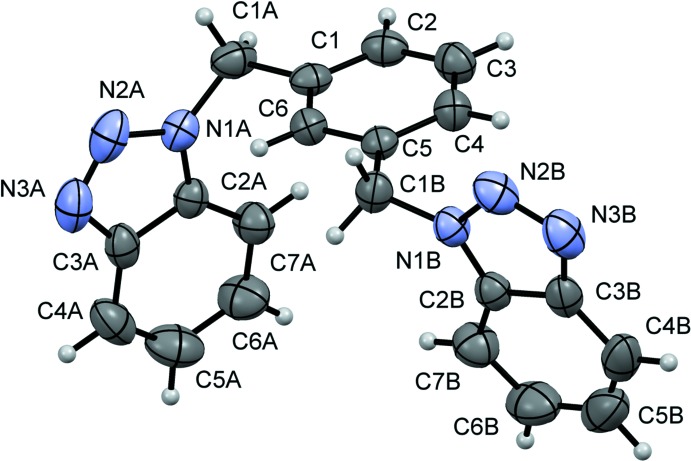
The mol­ecular structure of the title compound, showing anisotropic displacement ellipsoids drawn at the 50% probability level.

Fig. 1 ▸ shows the mol­ecule of the title compound. The mol­ecular structure is built by two benzotriazole groups describing a *meta* substitution of the central benzene ring. The dihedral angle between the two benzotriazole units is 57.08 (9)° and those between each benzotriazole moiety (N1–N3/C2–C7) and the central benzene ring are 88.74 (11) and 85.83 (10)° for the *A* and *B* groups, respectively. These values differ from the related structures 2,6-bis­(*N*,*N*′-benzotriazol-1-ylmeth­yl)pyridine, with a pyridine central ring, where the angle between the two benzotriazole units is 72.49 (6)° and those between the pyridine ring and the two benzotriazole units are 70.26 (6) and 57.70 (7)° (Selvanayagam *et al.*, 2002 ▸), and from the 1,4-bis­(1*H*-benzotriazol-1-ylmeth­yl)benzene tetra­hydrate, with *para* substitution, where the two benzotriazole units are parallel and the dihedral angle between each benzotriazole unit and the central benzene ring is 74.95 (9)° (Cai *et al.*, 2004 ▸).

## Supra­molecular features   

**Figure 2:**
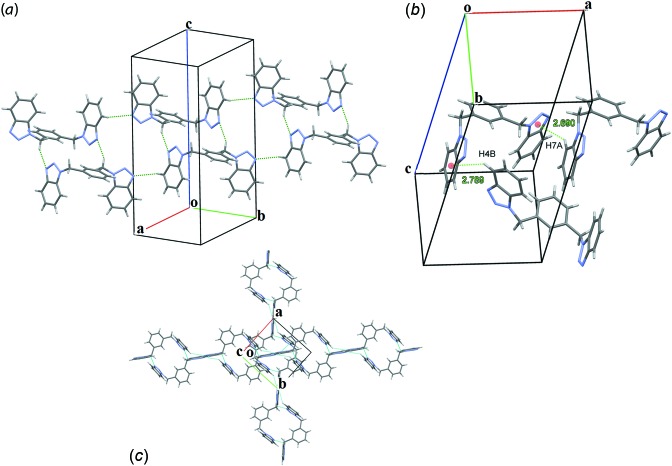
The crystal structure of the title compound showing the hydrogen-bond inter­actions: (*a*) C—H⋯N along [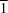
10], (*b*) C—H⋯π and (*c*) orthogonal chains viewed along [001].

The packing is directed by weak C—H⋯N and C—H⋯π inter­actions as shown in Table 1 ▸. Pairs of inversion-related mol­ecules, connected by two equivalent weak C1*B*—H1*BA*⋯N3*A* ^i^ [symmetry code: (i) −*x* + 1, −*y* + 1, −*z* + 1] inter­actions form slabs of infinite chains of mol­ecules running along [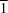
10]. Each mol­ecule in a slab connects to two translation-equivalent mol­ecules through C4*A*—H4*A*⋯N3*B* ^ii^ [symmetry code: (ii) *x* − 1, *y* + 1, *z*] inter­actions (Fig. 2 ▸ *a*). Parallel chains inter­act through C7*A*—H7*A*⋯*Cg*1^iii^ [*Cg*1 is the centroid of the N2*B*–N1*B*–C2*B*–C3*B*–N3*B* ring; symmetry code: (iii) 1 − *x*, *y*, *z*] (Fig. 2 ▸ *b*). Since the chains run along the diagonal of the *ab* plane and *a*≃*b*, the 2_1_ screw axis parallel to *b* transforms each chain into an orthogonal one, running along [110] (Fig. 2 ▸ *c*). This orthogonal chain inter­acts with the initial one through C4*B*—H4*B*⋯*Cg*2^iv^ [*Cg*2 is the centroid of the C4*A*-C3*A*-C2*A*-C7*A*-C6*A*-C5*A* ring; symmetry code: (iv) 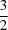 − *x*, −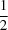 + *y*, 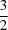 − *z*] (Fig. 2 ▸ *b*). In this way, each mol­ecule displays four pairs of inter­actions with seven neighbouring mol­ecules. This crystallographic three-dimensional organization differs from the related 1,4-bis­(1*H*-benzotriazol-1-ylmeth­yl)benzene tetra­hydrate where a two-dimensional network is observed (Cai *et al.*, 2004 ▸).

## Database survey   

A search of the Cambridge Structural Database (CSD Version 5.36 with one update; Groom *et al.*, 2016 ▸) for the 1,3-bis­(1*H*-benzotriazol-1-ylmeth­yl)benzene mol­ecular structure with the possibility of any group replacing the 2,4,5,6-H atoms in the central benzene ring gave four hits, from which two have one additional arene substituent (Br, -OCH_2_Ph), one has the bis­(1*H*-benzotriazole-1-yl­methanone) moiety instead of bis­(1*H*-benzotriazol-1-ylmeth­yl) and the last one corresponds to a more complex mol­ecular structure. When the search also considers heterocyclic compounds, two new hits (in addition to the first four structures) appear, one cyclic bi­pyridine and the related mol­ecular structure 2,6-bis­(1*H*-benzotriazol-1-yl­meth­yl)pyridine. A search for the 1,4-bis­(1*H*-benzotriazol-1-ylmeth­yl)-substituted benzene ring gave four hits, one of which corresponds to a ligand with additional methyl groups at the 1,3,5,6-sites of the central benzene ring and the other to its corresponding palladium complex. The remaining two relate to the same compound, *viz*. 1,4-bis­(1*H*-benzotriazol-1-yl­meth­yl)benzene tetra­hydrate, a related mol­ecular structure.

## Synthesis and crystallization   

*m*-Xylylene dibromide (1.16 g, 4.4 mmol) was added to a solution of 1*H*-benzotriazole (1.04 g, 8.7 mmol) in toluene (60 mL), and the mixture was heated at reflux for 72 h. The resulting mixture was filtered, and the toluene solution was concentrated and cooled to give a white solid. Single crystals suitable for X-ray structure analysis were obtained by dissolv­ing the compound in the minimum volume of di­chloro­methane, adding diethyl ether and cooling the solution to 277 K. The title compound formed colorless parallelepipeds. Yield: 668 mg (45%). M.p. 423–424 K. IR (KBr, cm^−1^): ν 3058 (*w*), 3031 (*w*), 2979 (*w*), 2944 (*w*), 1613 (*m*), 1494 (*m*), 1452 (*s*), 1228 (*s*), 1159 (*m*), 1080 (*s*), 781 (*s*), 754 (*s*), 743 (*s*). ^1^H NMR (400 MHz, DMSO-*d_6_*): δ (p.p.m.) 8.04 (*d*, *J* = 8.3 Hz, 2H), 7.72 (*d*, *J* = 8.3 Hz, 2H), 7.47 (*t*, *J* = 8.1 Hz, 2H), 7.38 (*t*, *J* = 8.2 Hz, 2H), 7.36 (*s*, 1H), 7.32 (*dd*, *J* = 8.5, 6.6 Hz, 1H), 7.25 (*d*, *J* = 8.2 Hz, 2H), 5.95 (*s*, 4H). ^13^C NMR (100 MHz, DMSO-*d_6_*): δ (p.p.m.) 145.3 (C), 136.5 (C), 132.6 (C), 129.3 (CH), 127.5 (CH), 127.4 (CH), 127.2 (CH), 124.0 (CH), 119.2 (CH), 110.6 (CH), 50.7 (CH_2_). HRMS *m*/*z* (ESI) calculated for [C_20_H_16_N_6_+H]^+^: 341.1509; found 341.1532 [*M*+H]^+^.

## Refinement   

Crystal data, data collection and structure refinement details are summarized in Table 2 ▸. H atoms were placed in calculated positions (C—H: 0.93–0.97 Å) and included as riding contributions with isotropic displacement parameters set at 1.2–1.5 times the *U*_eq_ value of the parent atom.

## Supplementary Material

Crystal structure: contains datablock(s) I. DOI: 10.1107/S2056989016007805/bg2585sup1.cif

Structure factors: contains datablock(s) I. DOI: 10.1107/S2056989016007805/bg2585Isup2.hkl

Supporting information file. DOI: 10.1107/S2056989016007805/bg2585Isup3.cml

CCDC reference: 1479416

Additional supporting information:  crystallographic information; 3D view; checkCIF report

## Figures and Tables

**Table 1:** Hydrogen-bond geometry (Å, °) *Cg*1 and *Cg*2 are the centroids of the N1*B*–N3*B*/C2*B*/C3*B* C2*A*–C7*A* rings, respectively.

*D*—H⋯*A*	*D*—H	H⋯*A*	*D*⋯*A*	*D*—H⋯*A*
C1*B*—H1*BA*⋯N3*A* ^i^	0.97	2.59	3.409 (4)	142
C4*A*—H4*A*⋯N3*B* ^ii^	0.93	2.66	3.443 (4)	143
C7*A*—H7*A*⋯*Cg*1^iii^	0.93	2.69	3.423 (3)	136
C4*B*—H4*B*⋯*Cg*2^iv^	0.93	2.89	3.481 (3)	132

Symmetry codes: (i) 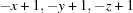; (ii) 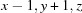; (iii) 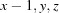; (iv) 
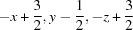
.

**Table 2:** Experimental details

Crystal data	
Chemical formula	C_20_H_16_N_6_
*M*_r_	340.39
Crystal system, space group	Monoclinic, *P*2_1_/*n*
Temperature (K)	293
*a*, *b*, *c* (Å)	9.3050 (5), 9.4479 (5), 19.5429 (9)
β (°)	99.205 (2)
*V* (Å^3^)	1695.94 (15)
*Z*	4
Radiation type	Mo *K*α
μ (mm^−1^)	0.08
Crystal size (mm)	0.40 × 0.39 × 0.18

Data collection	
Diffractometer	Bruker D8 Venture/Photon 100 CMOS
Absorption correction	Multi-scan (*SADABS*; Bruker, 2013 ▸)
*T*_min_, *T*_max_	0.666, 0.746
No. of measured, independent and observed [*I* > 2σ(*I*)] reflections	28764, 3480, 2830
*R*_int_	0.035
(sin θ/λ)_max_ (Å^−1^)	0.626

Refinement	
*R*[*F* ^2^ > 2σ(*F* ^2^)], *wR*(*F* ^2^), *S*	0.067, 0.160, 1.42
No. of reflections	3480
No. of parameters	236
H-atom treatment	H-atom parameters constrained
Δρ_max_, Δρ_min_ (e Å^−3^)	0.24, −0.27

Computer programs: *APEX2* and *SAINT* (Bruker, 2013 ▸), *SHELXS2014* (Sheldrick, 2008 ▸), *SHELXL2014* (Sheldrick, 2015 ▸) and *Mercury* (Macrae *et al.*, 2008 ▸).

## supplementary crystallographic information

### Crystal data

**Table:**

C_20_H_16_N_6_	*F*(000) = 712
*M**_r_* = 340.39	*D*_x_ = 1.333 Mg m^−^^3^
Monoclinic, *P*2_1_/*n*	Mo *K*α radiation, λ = 0.71073 Å
*a* = 9.3050 (5) Å	Cell parameters from 9846 reflections
*b* = 9.4479 (5) Å	θ = 3.0–27.1°
*c* = 19.5429 (9) Å	µ = 0.08 mm^−^^1^
β = 99.205 (2)°	*T* = 293 K
*V* = 1695.94 (15) Å^3^	Parallelepiped, colorless
*Z* = 4	0.40 × 0.39 × 0.18 mm

### Data collection

**Table:**

Bruker D8 Venture/Photon 100 CMOS diffractometer	3480 independent reflections
Radiation source: Mo sealed tube	2830 reflections with *I* > 2σ(*I*)
Detector resolution: 10.4167 pixels mm^-1^	*R*_int_ = 0.035
φ and ω scans	θ_max_ = 26.4°, θ_min_ = 3.0°
Absorption correction: multi-scan (*SADABS*; Bruker, 2013)	*h* = −11→11
*T*_min_ = 0.666, *T*_max_ = 0.746	*k* = −11→11
28764 measured reflections	*l* = −24→24

### Refinement

**Table:**

Refinement on *F*^2^	Hydrogen site location: inferred from neighbouring sites
Least-squares matrix: full	H-atom parameters constrained
*R*[*F*^2^ > 2σ(*F*^2^)] = 0.067	*w* = 1/[σ^2^(*F*_o_^2^) + (0.036*P*)^2^ + 0.9412*P*] where *P* = (*F*_o_^2^ + 2*F*_c_^2^)/3
*wR*(*F*^2^) = 0.160	(Δ/σ)_max_ < 0.001
*S* = 1.42	Δρ_max_ = 0.24 e Å^−^^3^
3480 reflections	Δρ_min_ = −0.27 e Å^−^^3^
236 parameters	Extinction correction: *SHELXL2014* (Sheldrick, 2015), Fc^*^=kFc[1+0.001xFc^2^λ^3^/sin(2θ)]^-1/4^
0 restraints	Extinction coefficient: 0.0162 (17)

### Special details

**Table:**

Geometry. All esds (except the esd in the dihedral angle between two l.s. planes) are estimated using the full covariance matrix. The cell esds are taken into account individually in the estimation of esds in distances, angles and torsion angles; correlations between esds in cell parameters are only used when they are defined by crystal symmetry. An approximate (isotropic) treatment of cell esds is used for estimating esds involving l.s. planes.

### Fractional atomic coordinates and isotropic or equivalent isotropic displacement parameters (Å^2^)

**Table:**

	*x*	*y*	*z*	*U*_iso_*/*U*_eq_	
C1	0.2899 (2)	0.1606 (3)	0.54469 (11)	0.0400 (5)	
C2	0.2736 (3)	0.0198 (3)	0.56154 (13)	0.0475 (6)	
H2	0.1814	−0.0207	0.5552	0.057*	
C3	0.3936 (3)	−0.0607 (3)	0.58775 (13)	0.0499 (6)	
H3	0.3820	−0.1554	0.5985	0.060*	
C4	0.5308 (3)	−0.0010 (3)	0.59803 (13)	0.0454 (6)	
H4	0.6109	−0.0560	0.6159	0.054*	
C5	0.5505 (2)	0.1396 (2)	0.58204 (11)	0.0366 (5)	
C6	0.4286 (2)	0.2197 (3)	0.55500 (12)	0.0401 (5)	
H6	0.4403	0.3141	0.5437	0.048*	
C1A	0.1582 (3)	0.2463 (3)	0.51371 (14)	0.0516 (7)	
H1AA	0.1547	0.2522	0.4639	0.062*	
H1AB	0.0708	0.1981	0.5223	0.062*	
N1A	0.1601 (2)	0.3890 (2)	0.54221 (10)	0.0433 (5)	
C2A	0.1399 (2)	0.4294 (2)	0.60655 (12)	0.0380 (5)	
N2A	0.1856 (3)	0.5036 (3)	0.50437 (13)	0.0606 (6)	
C1B	0.6995 (3)	0.2062 (3)	0.59004 (13)	0.0457 (6)	
H1BA	0.7311	0.2092	0.5451	0.055*	
H1BB	0.6932	0.3030	0.6059	0.055*	
N1B	0.8073 (2)	0.1311 (2)	0.63824 (10)	0.0408 (5)	
C3A	0.1528 (3)	0.5760 (3)	0.60636 (14)	0.0458 (6)	
N3A	0.1814 (3)	0.6171 (3)	0.54235 (14)	0.0621 (7)	
C2B	0.8262 (2)	0.1272 (2)	0.70871 (12)	0.0400 (5)	
N2B	0.9063 (2)	0.0455 (2)	0.61519 (12)	0.0530 (6)	
C4A	0.1384 (3)	0.6549 (3)	0.66532 (18)	0.0633 (8)	
H4A	0.1454	0.7531	0.6658	0.076*	
C3B	0.9426 (3)	0.0367 (3)	0.72765 (14)	0.0455 (6)	
N3B	0.9888 (2)	−0.0122 (2)	0.66848 (14)	0.0589 (6)	
C5A	0.1133 (4)	0.5806 (4)	0.72234 (18)	0.0716 (9)	
H5A	0.1039	0.6297	0.7626	0.086*	
C4B	0.9949 (3)	0.0093 (3)	0.79741 (17)	0.0656 (9)	
H4B	1.0748	−0.0492	0.8108	0.079*	
C6A	0.1013 (4)	0.4324 (4)	0.72188 (15)	0.0654 (8)	
H6A	0.0840	0.3864	0.7618	0.078*	
C5B	0.9239 (4)	0.0719 (4)	0.84490 (18)	0.0782 (11)	
H5B	0.9553	0.0550	0.8918	0.094*	
C7A	0.1143 (3)	0.3539 (3)	0.66457 (13)	0.0504 (6)	
H7A	0.1065	0.2557	0.6643	0.060*	
C7B	0.7529 (3)	0.1924 (3)	0.75700 (15)	0.0589 (7)	
H7B	0.6744	0.2529	0.7439	0.071*	
C6B	0.8042 (4)	0.1616 (4)	0.82488 (16)	0.0756 (10)	
H6B	0.7580	0.2015	0.8591	0.091*	

### Atomic displacement parameters (Å^2^)

**Table:**

	*U*^11^	*U*^22^	*U*^33^	*U*^12^	*U*^13^	*U*^23^
C1	0.0383 (12)	0.0479 (14)	0.0339 (11)	0.0014 (10)	0.0059 (9)	−0.0096 (10)
C2	0.0445 (13)	0.0528 (15)	0.0460 (14)	−0.0082 (12)	0.0102 (11)	−0.0078 (11)
C3	0.0537 (15)	0.0423 (14)	0.0545 (15)	−0.0078 (12)	0.0106 (12)	0.0013 (12)
C4	0.0477 (13)	0.0418 (13)	0.0466 (13)	0.0024 (11)	0.0074 (11)	0.0043 (11)
C5	0.0393 (12)	0.0386 (12)	0.0324 (11)	0.0002 (10)	0.0067 (9)	−0.0014 (9)
C6	0.0422 (12)	0.0378 (12)	0.0396 (12)	0.0009 (10)	0.0048 (10)	−0.0001 (10)
C1A	0.0419 (13)	0.0631 (17)	0.0472 (14)	0.0063 (12)	−0.0009 (11)	−0.0123 (12)
N1A	0.0405 (11)	0.0486 (12)	0.0407 (11)	0.0041 (9)	0.0059 (9)	0.0062 (9)
C2A	0.0316 (11)	0.0408 (12)	0.0414 (12)	0.0029 (9)	0.0052 (9)	0.0043 (10)
N2A	0.0552 (14)	0.0708 (17)	0.0561 (14)	0.0028 (12)	0.0096 (11)	0.0247 (13)
C1B	0.0420 (13)	0.0432 (13)	0.0502 (14)	−0.0009 (11)	0.0027 (11)	0.0070 (11)
N1B	0.0351 (10)	0.0399 (11)	0.0474 (11)	0.0040 (8)	0.0071 (8)	−0.0005 (9)
C3A	0.0370 (12)	0.0391 (13)	0.0600 (16)	0.0051 (10)	0.0041 (11)	0.0074 (11)
N3A	0.0586 (14)	0.0537 (14)	0.0725 (16)	0.0015 (12)	0.0062 (12)	0.0232 (13)
C2B	0.0373 (12)	0.0360 (12)	0.0466 (13)	−0.0016 (10)	0.0061 (10)	−0.0005 (10)
N2B	0.0445 (12)	0.0517 (13)	0.0651 (14)	0.0055 (10)	0.0155 (11)	−0.0103 (11)
C4A	0.0559 (17)	0.0421 (15)	0.091 (2)	0.0059 (13)	0.0083 (16)	−0.0118 (15)
C3B	0.0354 (12)	0.0368 (12)	0.0615 (16)	−0.0022 (10)	−0.0006 (11)	0.0035 (11)
N3B	0.0445 (12)	0.0496 (13)	0.0823 (17)	0.0103 (10)	0.0087 (12)	−0.0032 (12)
C5A	0.067 (2)	0.078 (2)	0.073 (2)	0.0010 (17)	0.0205 (16)	−0.0281 (18)
C4B	0.0582 (17)	0.0525 (17)	0.077 (2)	−0.0098 (14)	−0.0182 (15)	0.0150 (16)
C6A	0.077 (2)	0.072 (2)	0.0515 (16)	−0.0086 (17)	0.0232 (15)	−0.0035 (15)
C5B	0.100 (3)	0.071 (2)	0.0560 (19)	−0.028 (2)	−0.0110 (18)	0.0139 (17)
C7A	0.0571 (15)	0.0460 (14)	0.0490 (14)	−0.0057 (12)	0.0119 (12)	0.0020 (11)
C7B	0.0622 (17)	0.0583 (17)	0.0587 (17)	0.0045 (14)	0.0176 (14)	−0.0055 (14)
C6B	0.102 (3)	0.076 (2)	0.0519 (18)	−0.012 (2)	0.0221 (18)	−0.0090 (16)

### Geometric parameters (Å, º)

**Table:**

C1—C2	1.384 (4)	N1B—N2B	1.355 (3)
C1—C6	1.391 (3)	N1B—C2B	1.361 (3)
C1—C1A	1.513 (3)	C3A—N3A	1.376 (3)
C2—C3	1.380 (4)	C3A—C4A	1.397 (4)
C2—H2	0.9300	C2B—C3B	1.383 (3)
C3—C4	1.380 (4)	C2B—C7B	1.394 (4)
C3—H3	0.9300	N2B—N3B	1.310 (3)
C4—C5	1.384 (3)	C4A—C5A	1.369 (5)
C4—H4	0.9300	C4A—H4A	0.9300
C5—C6	1.395 (3)	C3B—N3B	1.376 (4)
C5—C1B	1.507 (3)	C3B—C4B	1.396 (4)
C6—H6	0.9300	C5A—C6A	1.404 (5)
C1A—N1A	1.457 (3)	C5A—H5A	0.9300
C1A—H1AA	0.9700	C4B—C5B	1.358 (5)
C1A—H1AB	0.9700	C4B—H4B	0.9300
N1A—N2A	1.354 (3)	C6A—C7A	1.365 (4)
N1A—C2A	1.355 (3)	C6A—H6A	0.9300
C2A—C3A	1.390 (3)	C5B—C6B	1.405 (5)
C2A—C7A	1.392 (3)	C5B—H5B	0.9300
N2A—N3A	1.308 (4)	C7A—H7A	0.9300
C1B—N1B	1.447 (3)	C7B—C6B	1.367 (4)
C1B—H1BA	0.9700	C7B—H7B	0.9300
C1B—H1BB	0.9700	C6B—H6B	0.9300
			
H1BA···N3A^i^	2.59	H7A···Cg1^iii^	2.69
H4A···N3B^ii^	2.66	H4B···Cg2^iv^	2.79
			
C2—C1—C6	119.1 (2)	N2B—N1B—C1B	120.9 (2)
C2—C1—C1A	119.8 (2)	C2B—N1B—C1B	129.5 (2)
C6—C1—C1A	121.1 (2)	N3A—C3A—C2A	108.2 (2)
C3—C2—C1	120.3 (2)	N3A—C3A—C4A	131.1 (3)
C3—C2—H2	119.8	C2A—C3A—C4A	120.7 (3)
C1—C2—H2	119.8	N2A—N3A—C3A	108.2 (2)
C2—C3—C4	120.2 (2)	N1B—C2B—C3B	104.9 (2)
C2—C3—H3	119.9	N1B—C2B—C7B	132.5 (2)
C4—C3—H3	119.9	C3B—C2B—C7B	122.6 (2)
C3—C4—C5	120.8 (2)	N3B—N2B—N1B	109.1 (2)
C3—C4—H4	119.6	C5A—C4A—C3A	116.7 (3)
C5—C4—H4	119.6	C5A—C4A—H4A	121.7
C4—C5—C6	118.5 (2)	C3A—C4A—H4A	121.7
C4—C5—C1B	122.0 (2)	N3B—C3B—C2B	108.6 (2)
C6—C5—C1B	119.4 (2)	N3B—C3B—C4B	130.7 (3)
C1—C6—C5	121.1 (2)	C2B—C3B—C4B	120.8 (3)
C1—C6—H6	119.4	N2B—N3B—C3B	107.8 (2)
C5—C6—H6	119.4	C4A—C5A—C6A	122.0 (3)
N1A—C1A—C1	112.5 (2)	C4A—C5A—H5A	119.0
N1A—C1A—H1AA	109.1	C6A—C5A—H5A	119.0
C1—C1A—H1AA	109.1	C5B—C4B—C3B	117.1 (3)
N1A—C1A—H1AB	109.1	C5B—C4B—H4B	121.5
C1—C1A—H1AB	109.1	C3B—C4B—H4B	121.5
H1AA—C1A—H1AB	107.8	C7A—C6A—C5A	122.0 (3)
N2A—N1A—C2A	110.1 (2)	C7A—C6A—H6A	119.0
N2A—N1A—C1A	121.6 (2)	C5A—C6A—H6A	119.0
C2A—N1A—C1A	128.3 (2)	C4B—C5B—C6B	121.5 (3)
N1A—C2A—C3A	104.7 (2)	C4B—C5B—H5B	119.2
N1A—C2A—C7A	132.7 (2)	C6B—C5B—H5B	119.2
C3A—C2A—C7A	122.6 (2)	C6A—C7A—C2A	116.0 (3)
N3A—N2A—N1A	108.8 (2)	C6A—C7A—H7A	122.0
N1B—C1B—C5	113.11 (19)	C2A—C7A—H7A	122.0
N1B—C1B—H1BA	109.0	C6B—C7B—C2B	115.5 (3)
C5—C1B—H1BA	109.0	C6B—C7B—H7B	122.3
N1B—C1B—H1BB	109.0	C2B—C7B—H7B	122.3
C5—C1B—H1BB	109.0	C7B—C6B—C5B	122.5 (3)
H1BA—C1B—H1BB	107.8	C7B—C6B—H6B	118.8
N2B—N1B—C2B	109.6 (2)	C5B—C6B—H6B	118.8

Symmetry codes: (i) −*x*+1, −*y*+1, −*z*+1; (ii) *x*−1, *y*+1, *z*; (iii) *x*−1, *y*, *z*; (iv) −*x*+3/2, *y*−1/2, −*z*+3/2.

### Hydrogen-bond geometry (Å, º)

*Cg*1 and *Cg*2 are the centroids of the N1*B*–N3*B*/C2*B*/C3*B* C2*A*–C7*A* rings, respectively.

**Table:**

*D*—H···*A*	*D*—H	H···*A*	*D*···*A*	*D*—H···*A*
C1*B*—H1*BA*···N3*A*^i^	0.97	2.59	3.409 (4)	142
C4*A*—H4*A*···N3*B*^ii^	0.93	2.66	3.443 (4)	143
C7*A*—H7*A*···*Cg*1^iii^	0.93	2.69	3.423 (3)	136
C4*B*—H4*B*···*Cg*2^iv^	0.93	2.89	3.481 (3)	132

Symmetry codes: (i) −*x*+1, −*y*+1, −*z*+1; (ii) *x*−1, *y*+1, *z*; (iii) *x*−1, *y*, *z*; (iv) −*x*+3/2, *y*−1/2, −*z*+3/2.
